# Case Report: Refractory hypokalemia as a clinical manifestation of Crooke’s cell adenoma

**DOI:** 10.3389/fmed.2025.1468727

**Published:** 2025-06-25

**Authors:** Laura Kattah, Lizeth Bustamante, Daniela Sanabria, Johana Salazar

**Affiliations:** ^1^Division of Endocrinology, Department of Internal Medicine, Hospital Universitario Fundación Santa Fe de Bogotá, Bogotá, Colombia; ^2^Department of Internal Medicine, Hospital Universitario Fundación Santa Fe de Bogotá, Bogotá, Colombia; ^3^Division of Endocrinology, Department of Internal Medicine, Universidad del Rosario, Bogotá, Colombia

**Keywords:** pituitary adenoma, ACTH, Crooke cell adenoma, Cushing disease, hypokalemia

## Abstract

Crooke cell adenomas (CCA) are a rare and aggressive subtype of corticotrope tumors, with a prevalence of less than 1% pituitary adenomas, commonly manifest as Cushing’s syndrome. We present the case of a 62-year-old male with progressive lower limb oedema, hypertension, and severe refractory hypokalemia. In this case, severe hypercortisolism was identified with the presence of a pituitary microadenoma of 9×6 mm and adrenal bilateral hyperplasia. Ectopic hypercortisolism was discarded after further evaluation. The patient was taken to a transsphenoidal endoscopic resection with complete resolution of symptoms. Histopathological and microscopic studies revealed findings consistent with Crooke cells compatible with Crooke cell adenoma. This tumor subtype exhibits a heterogeneous clinical presentation and is infrequently reported in the literature. Consequently, it represents an unpredictable clinical entity with a variable medical course.

## Introduction

The pituitary gland is a complex organ, with an evolving comprehension based on recent advances in pathology epigenetics and cell biology. The identification of different cell lineages and transcription factors and their role in pituitary cell differentiation and development has therefore been included in the most recent 2022 World Health Organization classification of central nervous system tumors. Particularly, a change in terminology from adenoma to pituitary neuroendocrine tumor (PitNET) has been proposed, providing a detailed subtyping of the tumor based on tumor cell lineage, cell type and related characteristics (1). Emphasis has been made on the expression of transcription factors (TF) and hormones, among other markers, using immunohistochemical staining to determine genealogical origin of tumor cells and adequate diagnosis (2). Classification amongst type is based on the staining of the main TFs which include PIT1, TPIT, SF-1, GATA3, and Erα, where TPIT, PIT1 and SF-1 define the major lineage PitNET type whereas the subtype highlights specific features within the framework of their normal cell counter parts. Importance has been placed on detecting aggressive subtypes of PitNETs that may warrant a more insistent medical approach whereas new subtypes have been introduced from previous general classification through specific differentiation. Although this classification has aimed on further specifying origin and cell development, limitations have been made due to the potential difficulties of adequate and permanent IHC staining in different scenarios as well as reproducibility. Additionally, histopathological findings do not correlate with clinical classification according to hormonal production and local symptoms which highlights the importance of further classifying every patient according to the clinical manifestations (2).

PIT1 cell lineage include somatotroph and lactotrophs tumors (with their respective subtypes classified as densely and sparsely granulated), thyrothroph tumor (no subtype) and PIT1 plurihormonal tumors which include mammosomatotroph tumor and acidophil stem cell tumor (recently introduced as distinct PIT1 subtypes from 2017 classification), mature and inmature PIT1 lineage tumor (previously known as PIT1-positive plurihormonal tumor) and mixed somatotroph and lactotroph tumor. SF1-lineage PitNETS include gonadotroph tumor (with no further subtype) whereas PitNETS with no distinct cell lineage are classified as plurihormonal tumor (multiple combinations) and null cel tumor (no documentation of TF) (2).

Corticotroph tumors responsible for the development of hypercortisolism and/or Cushing’s syndrome originate from the TPIT cellular lineage and are further subclassified into densely granulated, sparsely granulated, and Crooke cell adenomas (1, 2). Densely granulated forms resemble their normal cellular counterpart, usually manifesting with a more florid hormonal activity whereas sparsely granulated and crooke cell adenomas tend to be more aggressive in their clinical course. Crooke cell adenomas are rarely encountered, representing less than 1% of pituitary tumors and are composed of cells with hyaline or Crookes changes. These local changes would normally appear as evidence of negative hormonal feedback inhibition during hypercortisolism in non-neoplastic cells. However, these findings are not yet fully explained in Crookes adenomas. Furthermore, these tumors are amongst the most proliferative, aggressive, recurrent, and invasive PitNETs with the highest autonomous cortisol production described in the literature (3). Clinically, CCA can present as functional adenomas exhibiting hypercortisolism and Cushing syndrome as well as a hormonally silent incidental mass, with local compromise or even apoplexy (4). Treatment is surgical but it has a high risk of recurrence and even carcinomatous transformation (3, 5). We describe the first case of Crooke cell adenoma (CCA) presenting severe hypokalemia. Documenting clinical behavior during diagnosis and follow-up is essential for expanding the understanding of pituitary disorders and optimizing treatment options for this patient population.

## Case presentation

A 62-year-old man with a 6-month diagnosis of hypertension presented to the emergency department with 1 month of progressive lower limb oedema with no response to diuretic therapy. Current medications included telmisartan 40 mg QD, pill in the pocket with propafenone 60 mg, levothyroxine 175mcg 4 times a week and 150mcg 3 times a week, rosuvastatin 10 mg QD, tadalafil 5 mg QD, calcium citrate 1,500 mg QD and recently started, chlorthalidone 12.5 mg QD. Physical examination revealed high blood pressure of 150/85 mmHg with normal vital signs, weight of 92.2Kg (5 kg increase in 1 month) and BMI 28 kg/m^2^, grade III edema, and no other relevant findings. Medical history included paroxysmal atrial fibrillation, papillary thyroid carcinoma with remission criteria, and a recent decompression and arthrodesis of L4-L5, and L5-S1.

**Figure 1:**
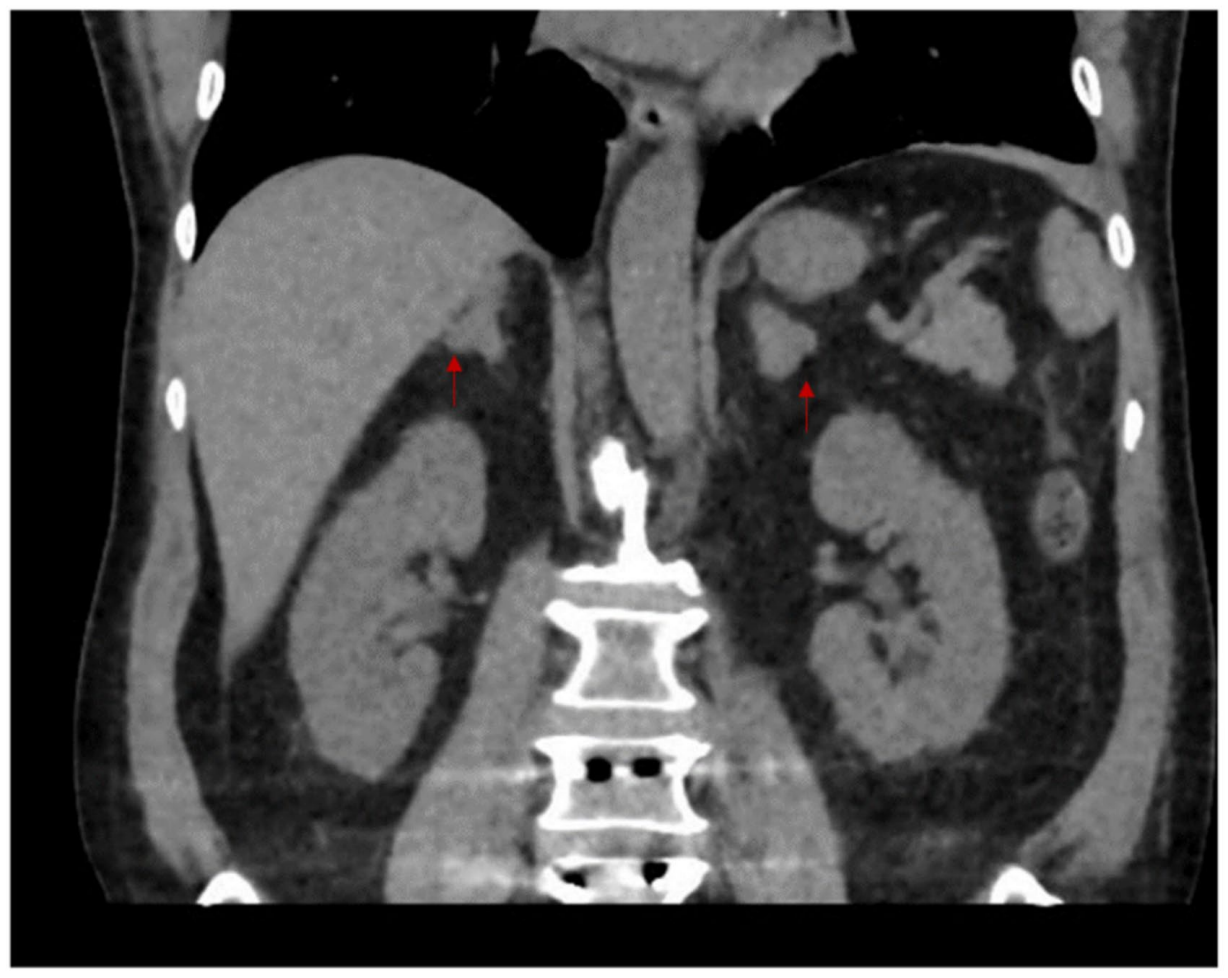
Abdominal CT scan. Arrows point to the adrenal glands. There is diffuse and fusiform thickening involving the medial and lateral arms, as well as the body of both adrenal glands, without any nodular lesions. In the clinical context of the patient, this finding suggests the consideration of adrenal hyperplasia.

**Figure 2:**
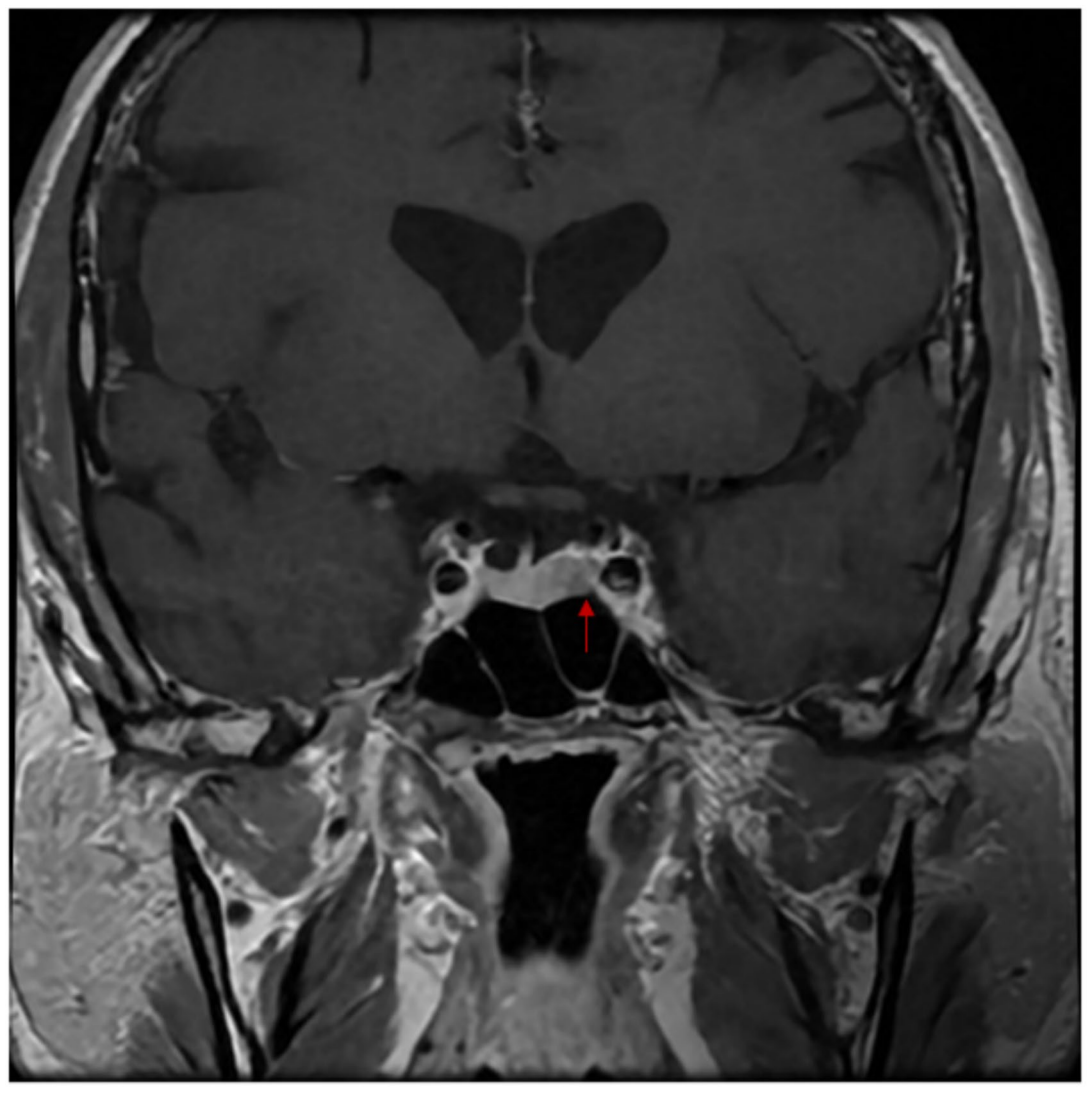
Magnetic resonance imaging of the sella turcica shows asymmetry in the adenohypophysis, with a greater size towards the left side. An ill-defined focal lesion, approximately 9×6 mm in size, is observed on the left side, which does not enhance with the contrast agent.

Blood tests revealed severe hypokalemia of 2.36 mEq/L (2.9 mmol/L) (RR: 3.5–5.0 mEq/L) with no other initial remarkable findings. Potassium infusion was initiated with titration up 16mEQ/h to maintain secure values. Abdominal CT showed diffuse, fusiform thickening of both adrenal glands compatible with bilateral adrenal hyperplasia (Figure 1). Additional studies revealed aldosterone 44.8 pg/ml (4.48 ng/dl) (RR: 25-392 pg/ml), DCR: 6.4 pg/ml (6 ng/L) (RR: 1-23 pg/ml), ARR: 1.6 (negative), UFC: 3107mcg/dl (85,710 nmol/L) (RR: 40-130mcg/dl) which was 30 times above the ULN, 8 am plasma cortisol: 5419 nmol/L (197mcg/dl) (RR: 185-624 nmol/L), overnight 1 mg DST: 214 nmol/L (7.7mcg/dl) with no suppression but a decrease of >50% and ACTH: 275.2 pg/ml (RR: 4.7–48.8 pg/ml). Pituitary MRI showed an ill-defined focal left lesion with no contrast enhancement of 6*9 mm (Figure 2). Complementary studies included normal thoracic and abdominal contrast CT scan, a non-diagnostic BIPPS and a negative octreotide scan. Although BIPSS was not confirmatory, central ACTH-dependent hypercortisolism was highly suspected after all the work-up. Throughout his hospital stay, he developed acute pulmonary thromboembolism involving the segmental and subsegmental branches to the upper lobe, left lower lobe, and lingula which required anticoagulation. This prolonged his surgical treatment.

**Figure 3:**
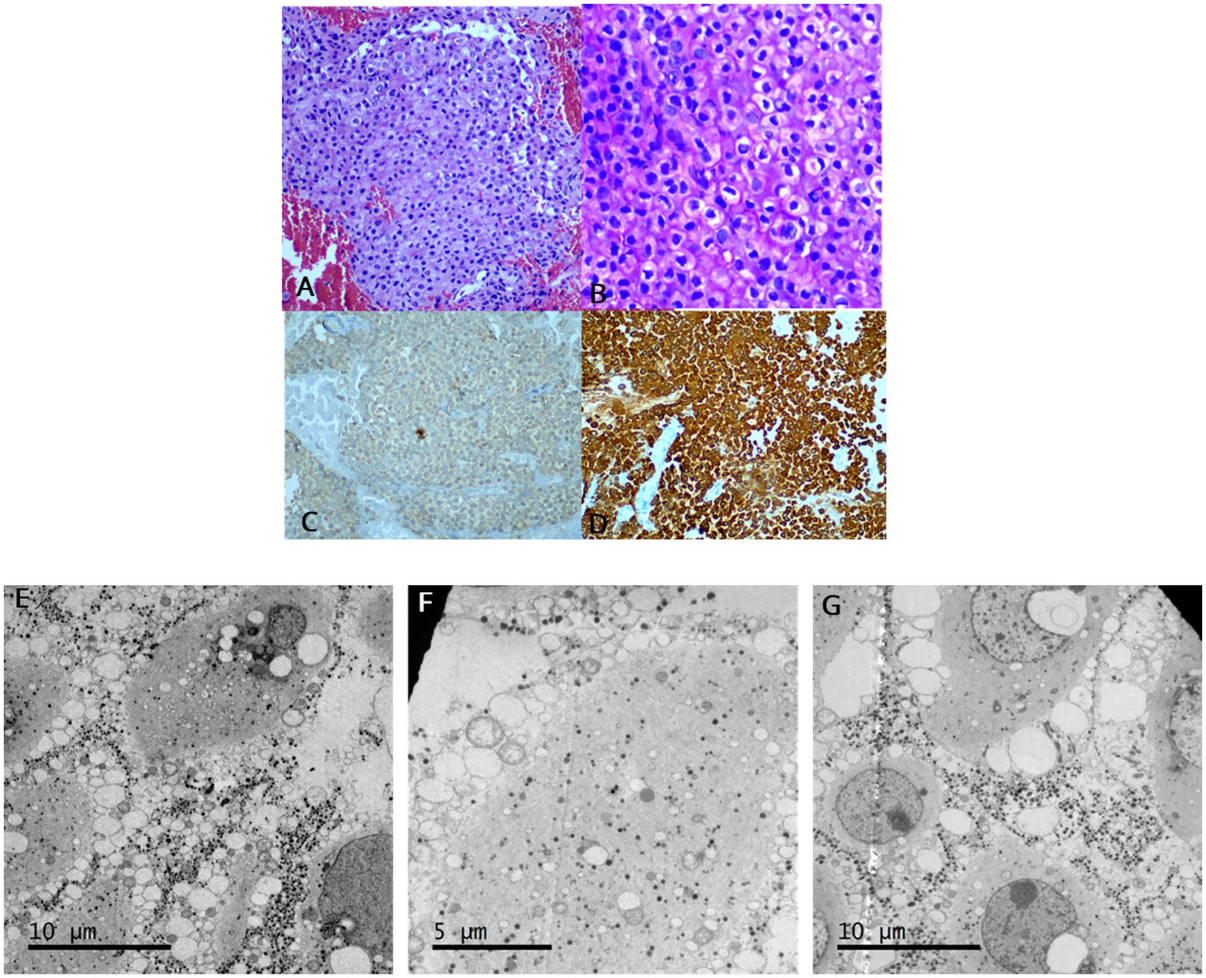
Surgical pathology examination and immunohistochemistry of the sellar mass from the case. Hematoxylin and eosin stain of the pituitary adenoma **(A)**, PAS staining highlights cell borders, which is unusual in other pituitary neuroendocrine tumors (40x) **(B)**. Adrenocorticotropin (ACTH) immunohistochemical stain showing many positive cells for ACTH **(C)**. CAM 5.2 immunohistochemical stain showing many positive cells for CAM 5.2 **(D)**. Ultrastructure study identified cells with the presence of small peripheral granules, cytoplasm-containing filaments, no evidence of the Golgi apparatus, and the presence of a dilated reticulum. The nuclei show prominent nucleoli **(E–G)**.

After confirmatory tests and considering the severe symptoms such as persistent hypokalemia, progressive hypertension, persistent edema and prothrombotic state, medical treatment was initiated with ketoconazole 200 mg twice a day which was titrated up to 1,200 mg a day, obtaining a decrease in cortisol level of 84% and a decrease in potassium infusion from 16 mEQ/h to 6 mEQ/h. He was eventually taken to transsphenoidal endoscopic resection with no complications and a favorable outcome with rapid normalization of potassium levels. Histopathological results displayed long basophilic cells with hyaline Crooke’s changes, positive immunohistochemical staining for ACTH, and ultrastructure images of cells with small peripheral granules and cytoplasm with filaments and no Golgi apparatus or dilated endoplasmic reticulum (Figure 3).

After surgical intervention, hypokalemia was completely resolved with progressive resolution of oedema and normalization of hypertension. Close monitoring of cortisol and ACTH levels demonstrated a progressive decrease. He was discharged with extended continued anticoagulant therapy considering a high risk of thromboembolic disease associated with hypercortisolism and neoplastic conditions, using direct anticoagulants. Low doses of hydrocortisone were used transiently for 4 weeks with posterior paraclinical evaluation at 3 months obtaining UFC 45mcg/24 hours (1,215 nmol) (RR: 40-130mcg/dl). After 2 years of follow up and intervention, he has normal cortisol and ACTH values, with increasing but in range UFC (Table 1). No recurrence changes were evident in the pituitary MRI and abdominal CT, which showed a decrease in adrenal hyperplasia. He remains asymptomatic.

**Table 1:** Behavior of serum cortisol, ACTH, urinary free cortisol (UFC), 11 pm salivary cortisol, cortisol post 1 mg of dexamethasone and potassium from diagnosis, medical treatment, post-surgical treatment and up to 24 months of follow-up.

Biochemical parameters/Clinical course	Diagnosis	Post Ketoconazole initiation	Pre-surgical	Post-surgical (72 h)	Follow-up (months)				
					3	12	15	18	24
Plasma cortisol (mcg/dl)	197	39,8	37,73	15,26	9,6	11,74		5,85	7,3
Urinary free cortisol (UFC) (mcg/24 h)^a^	3,125	1,528			45	114	122	143	24
Cortisol post 1 mg dexamethasone (mcg/dl)	7,7								
11 pm salivary cortisol (nmol/l) ^b^					2,37	3,03	2,53		2,28
ACTH (pg/ml) ^c^	275	177		15,14	18,51	6,29		5,73	9,14
Potassium (mEq/L)	2,36 ^d^	3,8 ^e^		4, 0 ^f^	4,28 ^f^	4,45 ^f^	4,68 ^f^	4,5 ^f^	4,46 ^f^

Reference level ^a^ (6,72–22,69 mcg/24 h), ^b^ (<5,4), ^c^ (4.7–48.8 pg/ml). Potassium levels in relation to potassium administration ^d^ 16mEQ/L K + infusion ^e^ 6mEQ/L K + infusion ^f^ no k + infusion.

## Discussion

Crooke cell adenomas represent a rare cause of pituitary tumors with a prevalence of less than 1%. To this date, fewer than 150 cases have been reported, demonstrating a heterogeneous behavior and a variable clinical course (3, 6). Approximately 65% of cases present with Cushing syndrome with a wide spectrum of manifestations. However, to our knowledge, this is the first case reported in the literature of severe hypokalemia associated with hypertension and edematous syndrome as the primary manifestation of CCA (7).

In normal conditions, the mineralocorticoid receptor (MR) is primarily activated by aldosterone even though cortisol can bind and activate the receptor as well. To ensure an appropriate regulation of the receptors activity, cortisol is converted to cortisone by the enzyme 11β-hydroxysteroid dehydrogenase type 2 (11BHSD2) which has a very low affinity for the MR ensuing no activation, a process known as the cortisol—cortisone shunt (8). However, excess ACTH and secondary hypercortisolism lead to saturation of the enzyme’s activity and the subsequent activation of the MR leading to hypertension and potassium wasting. ACTH also increases the production of other weak mineralocorticoids such as 11-deoxicorticosterone, and it augments the activity of the renin—angiotensin—aldosterone system amongst other mechanisms which can further cause hypertension and hypokalemia (9). This type of manifestation is more common in ectopic ACTH-secreting tumors and is seldom described in pituitary tumors (10). The largest published cohort of CCA describes the highest prevalence amongst women with a majority of macroadenomas as opposed to our case in which we describe a male patient with a microadenoma (3).

CCA treatment is surgical with a relapse rate of 50% and a 9% risk of metastasis development. Follow-up studies are scarce but mostly demonstrate aggressive tumor behavior with a low rate of surgical success after relapse and are often unresponsive to medical or radiotherapy protocols (11). Gamma knife radiotherapy has been recently described as an adjuvant therapy in a four-patient series with acceptable results (5). Temozolomide (TMZ), an oral alkylating agent used predominantly in the treatment of glioblastoma has shown efficacy in the salvage treatment of aggressive pituitary tumors since 2006 onwards, through its mechanism of action of alkylating DNA bases (predominantly guanine) and inducing DNA fragmentation by repairing enzymes in such locations (11–13). Reports of long-term remission describe a low expression of O-6-methylguanine-DNA methyltransferase (MGMT) a DNA repair enzyme commonly found in invasive corticotroph adenomas including CCA which has been noted to predict TMZ responsiveness (14–17).

Histologically, CCA tumors are characterized by having more than 50% hyaline Crooke cells which are described as large, spheric, or ovoid, basophilic cells with PAS and ACTH positive granules sequestered at the cell’s periphery or around the nucleus. Conversely, the cytoplasm is filled with a ring of hyaline material which is intensely reactive to keratins (CKD8, CAM5.2, and AE1/AE3). This accumulated material decreases the quantity of cytosolic organelles as the Golgi apparatus and the rough endoplasmic reticulum (1).

Crooke change is a phenomenon of pituitary cells as a response to excess cortisol, such as a morphological manifestation of functional suppression. The extent of the changes may be an indicator of the degree of ACTH suppression of normal corticotropes as demonstrated by Oldfield et al., where a positive correlation was described between the hyaline changes and UFC, specifically when were 4 times above the normal value (18). However, in CCA it is not fully understood why adenoma cells produce ACTH and still develop crooks’ hyaline changes in response to glucocorticoid excess. It was originally thought that these changes were secondary to receptor overexpression or hypersensitivity. Nonetheless, not all CCA are associated with hypercortisolism which suggests other pathophysiologic mechanisms (11, 19).

As previously described, CCA has a heterogeneous presentation which may explain the severe hypercortisolism our patient presented with a small non-invasive tumor. To this date, our patient remains asymptomatic, with increasing but in range UFC, ACTH below 10 pg/ml and no imagological findings but follow-up has only been 24 months. Strict follow up remains mandatory. To our knowledge, this is the first case of severe hypokalemia associated with ACC. This further elucidates the diversity of symptoms and spectrum of disease associated with Cushing syndrome.

## Data Availability

The raw data supporting the conclusions of this article will be made available by the authors, without undue reservation.
