# Supplementary material for: Case Report: Refractory hypokalemia as a clinical manifestation of Crooke’s cell adenoma
Source: Front Med (Lausanne). 2025 Jun 25;12:1468727. doi: 10.3389/fmed.2025.1468727 (PMC12238053; doi:10.3389/fmed.2025.1468727)
Supplement: Supplementary file 1 [file Data_Sheet_1.pdf]

CARE Checklist of information to include when writing a case report

CARE case report guidelines

(cc) BY-NC-ND

Topic	Item	Checklist item description	Reported on Line
Title	1	The diagnosis or intervention of primary focus followed by the words "case report"	Yes Line 1
Key Words	2	2 to 5 key words that identify diagnoses or interventions in this case report, including "case report"	Yes 34
Abstract (no references)	3a	Introduction: What is unique about this case and what does it add to the scientific literature?	Yes 29-32 , 60-63
	3b	Main symptoms and/or important clinical findings	Yes 25-29, 60-61
	3c	The main diagnoses, therapeutic interventions, and outcomes	Yes 28-29
	3d	Conclusion—What is the main "take-away" lesson(s) from this case?	Yes 61-63
Introduction	4	One or two paragraphs summarizing why this case is unique (**may include references**)	Yes 61-63
Patient Information	5a	De-identified patient specific information.	Yes 66
	5b	Primary concerns and symptoms of the patient.	Yes 66-68
	5c	Medical, family, and psycho-social history including relevant genetic information	Yes 68-70
	5d	Relevant past interventions with outcomes	Yes 68-70
Clinical Findings	6	Describe significant physical examination (PE) and important clinical findings.	Yes 68-70
Timeline	7	Historical and current information from this episode of care organized as a timeline	No
Diagnostic Assessment	8a	Diagnostic testing (such as PE, laboratory testing, imaging, surveys).	Yes 72-79
	8b	Diagnostic challenges (such as access to testing, financial, or cultural)	Yes 80-81
	8c	Diagnosis (including other diagnoses considered)	Yes 79
	8d	Prognosis (such as staging in oncology) where applicable	No
Therapeutic Intervention	9a	Types of therapeutic intervention (such as pharmacologic, surgical, preventive, self-care)	Yes 106-109
	9b	Administration of therapeutic intervention (such as dosage, strength, duration)	Yes 136
	9c	Changes in therapeutic intervention (with rationale)	Yes 106-108
Follow-up and Outcomes	10a	Clinician and patient-assessed outcomes (if available)	Yes 132-135
	10b	Important follow-up diagnostic and other test results	Yes 132-135
	10c	Intervention adherence and tolerability (How was this assessed?)	No
	10d	Adverse and unanticipated events	yes 81-84
Discussion	11a	A scientific discussion of the strengths AND limitations associated with this case report	Yes 189-192
	11b	Discussion of the relevant medical literature **with references**.	Yes 146-192
	11c	The scientific rationale for any conclusions (including assessment of possible causes)	Yes 188-189
	11d	The primary "take-away" lessons of this case report (without references) in a one paragraph conclusion	Yes 188-192
Patient Perspective	12	The patient should share their perspective in one to two paragraphs on the treatment(s) they received	No
Informed Consent	13	Did the patient give informed consent? Please provide if requested	**Yes** ☒ **No** ☐
